# Supplementary material for: Peeping into Mitochondrial Diversity of Andaman Goats: Unveils Possibility of Maritime Transport with Diversified Geographic Signaling
Source: Genes (Basel). 2023 Mar 24;14(4):784. doi: 10.3390/genes14040784 (PMC10138289; doi:10.3390/genes14040784)
Supplement: Supplementary file 1 [file genes-14-00784-s001.zip › genes-2083685-supplementary.pdf]

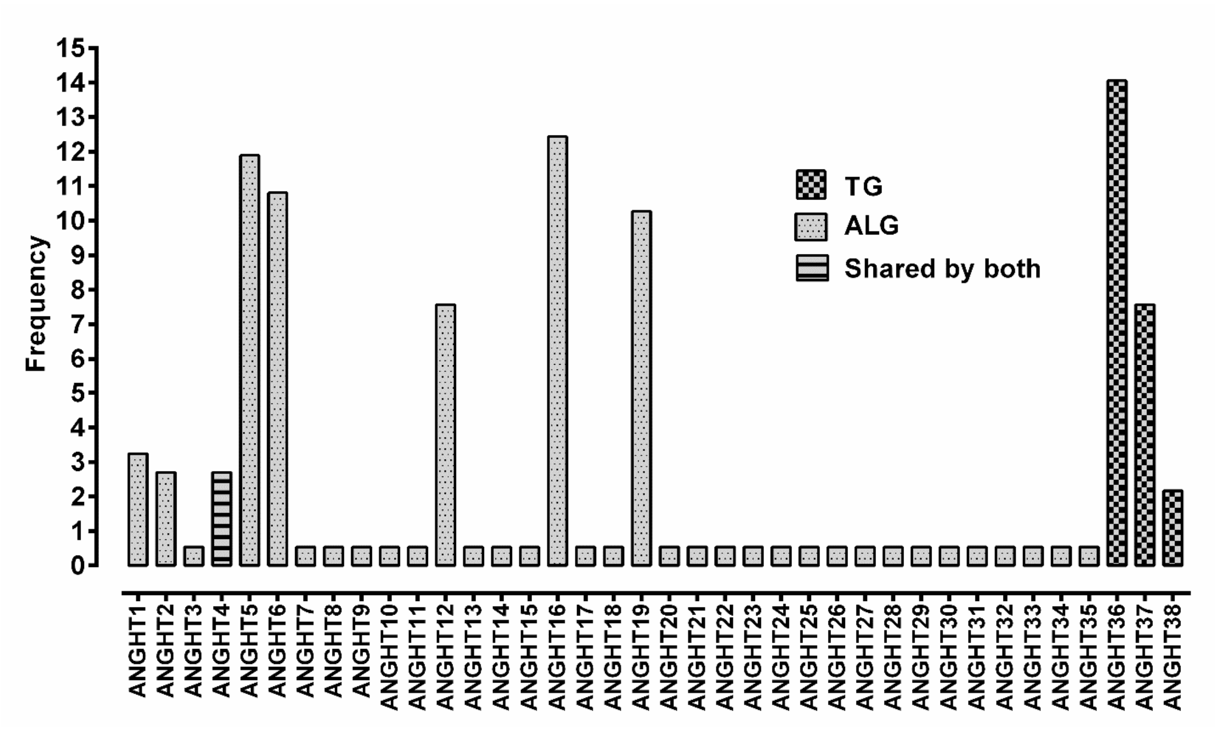

Figure S1. Frequency of the detected haplotypes of Andaman goats. ANC indicates Andaman goat, TG indicates Teressa goat and HT indicates haplotypes.

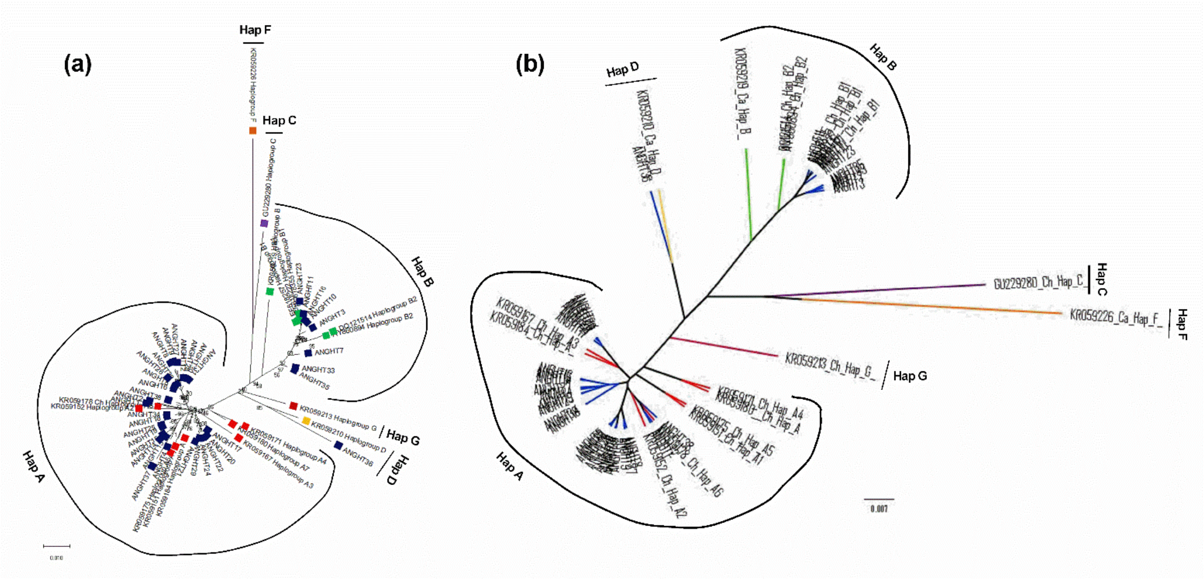

Figure S2. **Haplogroup assignment of different haplotypes of Andaman goat.** (**a**) Neighbor Joining (NJ) phylogenetic tree, (**b**) Bayesian phylogenetic tree. The GenBank accession numbers of the standard haplogroups used are as follows; A=KR059184, KR059151, KR059152, KR059167, KR059171, KR059175, KR059178, KR059180; B=KR059219, EF618257, EF618222, EF618355, DQ121514, AY860894; C=GU229280; D=KR059210; F=KR059226 and G=KR059213. NJ tree was drawn in MEGAX [27] and Bayesian phylogenetic tree was drawn in BEAST v1.10.4 [32]. The phylogenetic trees and network map were constructed based on 481 bp hypervariable region (HV1) of goat mitochondrial D-loop.

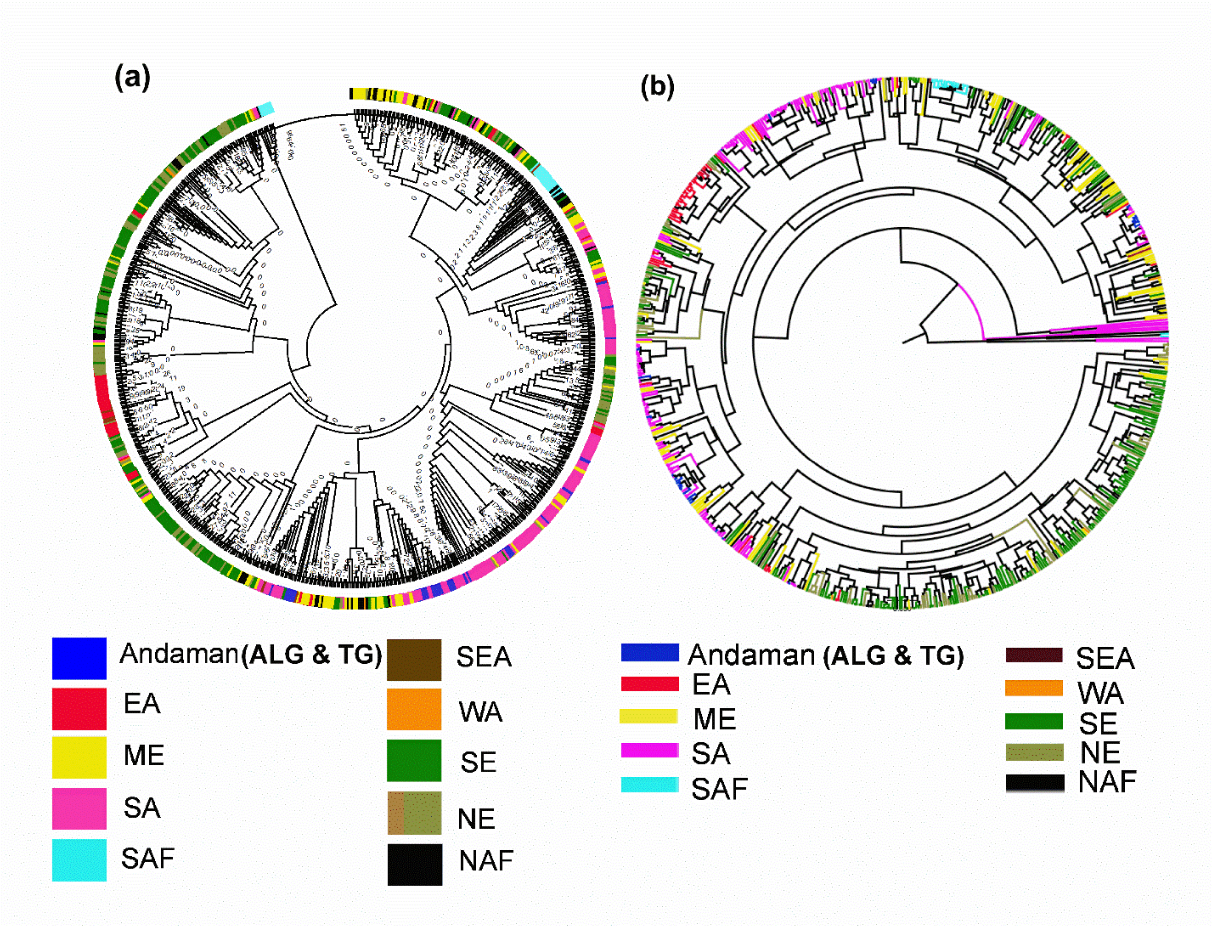

Figure S3. Haplogroup A of Andaman goats and its relationship with A haplogroup goats of different regions (**a**) Neighbor Joining (NJ) phylogenetic tree, (**b**) Bayesian phylogenetic tree. The Neighbor-Joining tree was constructed using Tamura-Nei model implemented in MEGAX [28] following 1,000 bootstrap replications, the Bayesian phylogenetic tree was drawn in BEAST v1.10.4 [33]. Analysis was done based on 481 bp hypervariable region (HV1) of mitochondrial D-loop.

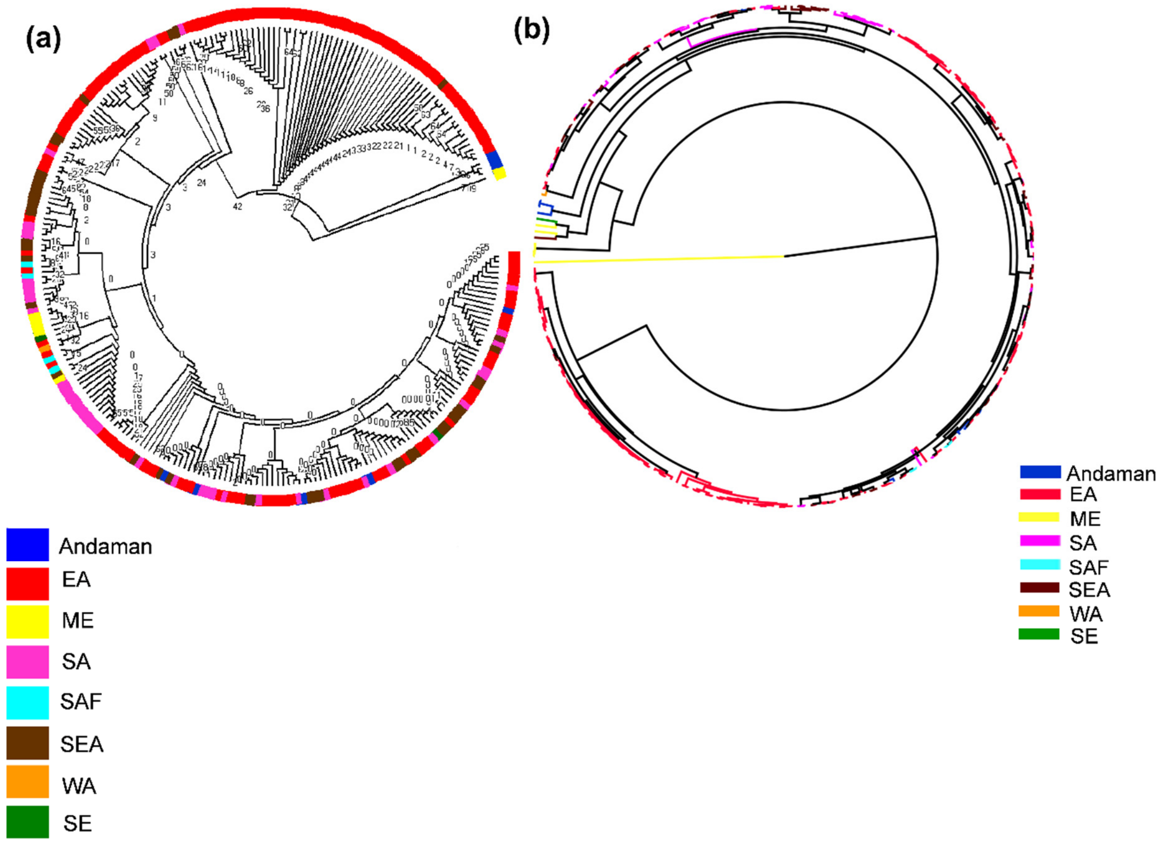

Figure S4. Haplogroup B of Andaman goats and its relationship with B haplogroup goats of different regions (**a**) Neighbor Joining (NJ) phylogenetic tree. (**b**) Bayesian phylogenetic tree. The Neighbor-Joining tree was constructed using Tamura-Nei model implemented in MEGAX [27] following 1,000 bootstrap replications and the Bayesian phylogenetic tree was drawn in BEAST v1.10.4 [32]. Analysis was done based on 481 bp hypervariable region (HV1) of mitochondrial D-loop.

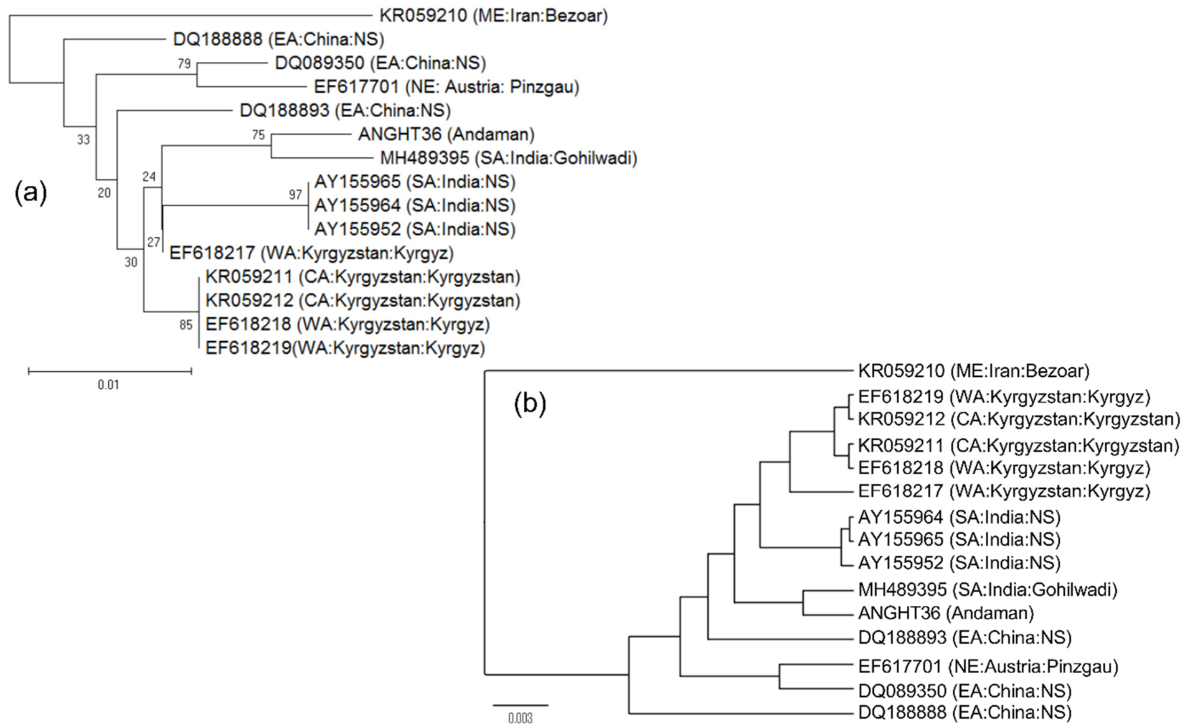

Figure S5. Haplogroup D of Andaman goats and its relationship with D haplogroup goats of different regions (a) Neighbor Joining (NJ) phylogenetic tree. **(b)** Bayesian phylogenetic tree. The Neighbor-Joining tree was constructed using Tamura-Nei model implemented in MEGAX [27] following 1,000 bootstrap replications and the Bayesian phylogenetic tree was drawn in BEAST v1.10.4 [32]. Analysis was done based on 481 bp hypervariable region (HV1) of mitochondrial D-loop.

Table S1. Sampling details of Andaman goats

S. No.	Sampling Stations	Latitude	Longitude	Sampling No.
**Andaman local goat**				
North & Middle Andaman (n=92)				
1.	Shyam Nagar	92.93593473 N	13.38970328 E	3
2.	Radha Nagar	92.94638363 N	13.37566189 E	3
3.	Swarajgram	92.91562781 N	13.32042218 E	3
4.	Laxmipur	92.97373775 N	13.28245757 E	3
5.	Kishori Nagar	92.86566866 N	13.24380604 E	3
6.	Madhupur	92.97272349 N	13.25591008 E	3
7.	Keralapuram	92.97272349 N	13.25591008 E	3
8.	Ramakrishnagram	92.96659232 N	13.25825047 E	3
9.	Durgapur	93.01117534 N	13.26017679 E	3
10.	Krishnapuri	92.95276374 N	13.2444902 E	3
11.	Subashgram	92.96265825 N	13.23913419 E	3
12.	Paranghara	92.89919957 N	13.21459469 E	3
13.	Nabagram	92.95365052 N	13.2117868 E	3
14.	Kalighat	92.95693449 N	13.12605033 E	3
15.	Danpur	92.90596207 N	12.90648728 E	3
16.	Lucknow	92.8955822 N	12.85602022 E	3
17.	Devpur	92.88448049 N	12.8546306 E	3
18.	Rampur	92.91675028 N	12.85694329 E	3
19.	Webi	92.89332767 N	12.83440262 E	3
20.	Pahalgaon	92.87488235 N	12.83234741 E	3
21.	Tugapur	92.85392245 N	12.83489173 E	3
22.	Nimbudera	92.88582924 N	12.7228287 E	3
23.	Billiground	92.89806781 N	12.67872744 E	3
24.	Dharmapur	92.90794967 N	12.64394107 E	3
25.	Kaushalyanagar	92.8843911 N	12.5459841 E	3
26.	Rangat	92.92169534 N	12.51202038 E	3
27.	Bharatpur	92.8921056 N	12.48814084 E	3
28.	Bakultala	92.86766631 N	12.5011245 E	3
29.	Kadamtala	92.79913261 N	12.34046686 E	3
30.	Adazig	92.7885048 N	12.2560715 E	2
31.	Kanchangarh	92.79272433 N	12.18314678 E	3
South Andaman (n=45)				
32.	Wimberlygunj	92.70833817 N	11.74389419 E	3
33.	Mathura	92.69320865 N	11.72619429 E	3
34.	Ferrargunj	92.66118942 N	11.72057477 E	3

35.	Bombooflat	92.68404218 N	11.693658 E	3
36.	Tusnabad	92.64995016 N	11.66952869 E	3
37.	Bindraban	92.67145743 N	11.71579361 E	3
38.	Chouldari	92.66202795 N	11.63791107 E	3
39.	Wandoor	92.66623221 N	11.6041036 E	3
40.	Manglutan	92.66366371 N	11.58205794 E	3
41.	Calicut	92.71995135 N	11.59747988 E	3
42.	Kamaraj Nagar	92.72601824 N	11.59948411 E	3
43.	Prothrapur	92.73776512 N	11.63014421 E	3
44.	Nayashahar	92.68688682 N	11.5798304 E	3
45.	Guptapara	92.66689237 N	11.55928596 E	3
46.	Manjery	92.66030049 N	11.54659548 E	3
**Teressa goats: Nicobar group of Islands (n=48)**				
1.	Aloorang	93.11495699 N	8.330705395 E	4
2.	Bengali	93.12078675 N	8.301399069 E	4
3.	Haoin	93.10937168 N	8.312291825 E	4
4.	Kalasi	93.11045884 N	8.289830662 E	4
5.	Minyuk	93.13292739 N	8.263474981 E	4
6.	Luxi teressa	93.15034308 N	8.225369793 E	4
7.	Katchal	93.38357789 N	8.011998694 E	4
8.	Kapanga	93.39753987 N	7.994153731 E	4
9.	Sonomkuwa	93.39375703 N	7.997173408 E	4
10.	Hintona	93.3983573 N	7.916490285 E	4
11.	Kamorta	93.49029766 N	8.170999007 E	4
12.	Champin	93.55425269 N	8.01995522 E	4

Table S2. Sequences used in the present study

Accession No.	Region	Country	Breed
**Haplogroup A**			
MK139101-02, MK139104-07, MK139109-10, MK139113-16, MK139118-25, MK139128-30, MT747101-26, MT747030-79 MN865073-79, MN865081	ISEA	Andaman	Andaman local goat
MK139132, MT747147-59, MK139136-37, MK139140, MT747160-64	ISEA	Andaman	Teressa Goat
AJ317545-52	EA	Mongolia	ND
DQ217780-85	EA	South Korea	ND
AY860871-80	EA	China	ND
AY860882-87	EA	China	ND
AY860893-93	EA	China	ND
AY860895-98	EA	China	ND
AY860900	EA	China	ND
AY860902	EA	China	ND
AY860905-06	EA	China	ND
AY860908-13	EA	China	ND
AY860915-16	EA	China	ND
AY860919-21	EA	China	ND
AY860923	EA	China	ND
AY860925-32	EA	China	ND
AY860934-42	EA	China	ND
DQ188849-56	EA	China	ND
DQ188858-59	EA	China	ND
DQ188861-72	EA	China	ND
DQ188874-77	EA	China	ND
DQ188879-85	EA	China	ND
DQ188887	EA	China	ND
DQ188889	EA	China	ND
DQ188894-95	EA	China	ND
DQ188897-903	EA	China	ND
DQ121491-99	EA	China	ND
DQ121501-03	EA	China	ND
DQ121505-13	EA	China	ND
DQ121518-27	EA	China	ND
DQ121531-34	EA	China	ND

DQ121536	EA	China	ND
DQ121540-41	EA	China	ND
DQ121543-48	EA	China	ND
DQ121550-64	EA	China	ND
DQ121568	EA	China	ND
DQ121572-77	EA	China	ND
DQ121580	EA	China	ND
DQ121582-84	EA	China	ND
DQ121586-90	EA	China	ND
DQ121592-96	EA	China	ND
DQ121599-609	EA	China	ND
DQ121613-15	EA	China	ND
DQ121617-18	EA	China	ND
AY853278-86	EA	China	ND
AY853288-90	EA	China	ND
AY853293	EA	China	ND
AY853296-99	EA	China	ND
DQ089106-11	EA	China	ND
DQ089113-14	EA	China	ND
DQ089116-17	EA	China	ND
DQ089119-23	EA	China	ND
DQ089125-28	EA	China	ND
DQ089130-33	EA	China	ND
DQ089135-37	EA	China	ND
DQ089139-48	EA	China	ND
DQ089151-53	EA	China	ND
DQ089155-57	EA	China	ND
DQ089161	EA	China	ND
DQ089164-75	EA	China	ND
DQ089177-78	EA	China	ND
DQ089180	EA	China	ND
DQ089182	EA	China	ND
DQ089186	EA	China	ND
DQ089189-90	EA	China	ND
DQ089194	EA	China	ND
DQ089198	EA	China	ND
DQ089200	EA	China	ND
DQ089202-04	EA	China	ND
DQ089206	EA	China	ND
DQ089208	EA	China	ND
DQ089210-13	EA	China	ND
DQ089216-17	EA	China	ND
DQ089219-22	EA	China	ND
DQ089224-25	EA	China	ND
DQ089228	EA	China	ND
DQ089230	EA	China	ND
DQ089232-34	EA	China	ND
DQ089236-39	EA	China	ND

DQ089242-44	EA	China	ND
DQ089246-48	EA	China	ND
DQ089250	EA	China	ND
DQ089252-55	EA	China	ND
DQ089259	EA	China	ND
DQ089262-63	EA	China	ND
DQ089265-66	EA	China	ND
DQ089271	EA	China	ND
DQ089274-75	EA	China	ND
DQ089277	EA	China	ND
DQ089280-82	EA	China	ND
DQ089284	EA	China	ND
DQ089286-94	EA	China	ND
DQ089297-99	EA	China	ND
DQ089303	EA	China	ND
DQ089309-49	EA	China	ND
DQ089351-56	EA	China	ND
DQ089359-62	EA	China	ND
DQ089365	EA	China	ND
DQ089367-68	EA	China	ND
DQ089370-73	EA	China	ND
DQ089375-76	EA	China	ND
DQ089379-95	EA	China	ND
DQ089397-402	EA	China	ND
DQ089406	EA	China	ND
DQ089409	EA	China	ND
DQ089414-20	EA	China	ND
DQ089422-59	EA	China	ND
DQ089462-73	EA	China	ND
AJ317569-70	EA	China	ND
EF618234-39	EA	Mongolia	ND
AJ317762-68	ME	Iraq	ND
AJ317769-73	ME	Jordan	ND
KR059184	ME	Saudi Arabia	ND
AJ317760-61	ME	Syria	ND
AJ317736-51	ME	Turkey	ND
AJ317842-43	ME	Turkey	ND
AJ317752-59	ME	Saudi Arabia	ND
KR059153	ME	Jordan	Baladie
KR059154	ME	Saudi Arabia	Najrani
KR059158	ME	Turkey	Hair
KR059160	ME	Jordan	Baladie
KR059161	ME	Turkey	Gurku
KR059179	ME	Turkey	Abaza
KR059183	ME	Jordan	Baladie
KR059184	ME	Saudi_Arabia	Beshi
KR059186	ME	Turkey	Abaza
KR059189	ME	Iran	Kermanshah

KR059191	ME	Iran	Kurdi
KR059192	ME	Iran	Naini
KR059199	ME	Turkey	Abaza
KR059200-02	ME	Turkey	Angora
KR059203	ME	Turkey	Gurku
KR059204	ME	Turkey	Hair
KR059206	ME	Iran	Taleshi
KR059207-08	ME	Iran	Kermanshah
KR059253-66	ME	Iran	Native Abadeh
KR059503	ME	Iran	Native Qazvin
KR059504	ME	Iran	Taleshi
KR059505	ME	Iran	Kuhaki
KR059506	ME	Iran	Turki
KR059507	ME	Iran	Native Qazvin
KR059508-11	ME	Iran	Taleshi
KR059512	ME	Iran	Khalkhali
KR059513	ME	Iran	Turki
KR059514	ME	Iran	Native Qazvin
KR059515-16	ME	Iran	Taleshi
KR059517	ME	Iran	Kuhaki
KR059518	ME	Iran	Turki
KR059519	ME	Iran	Native Qazvin
KR059520	ME	Iran	Turki
KR059521	ME	Iran	Native Qazvin
KR059522-23	ME	Iran	Taleshi
KR059524	ME	Iran	Kuhaki
KR059525	ME	Iran	Naini
KR059526	ME	Iran	Turki
KR059527	ME	Iran	Native Qazvin
KR059528	ME	Iran	Turki
KR059529	ME	Iran	Kuhaki
KR059530-31	ME	Iran	Turki
KR059532	ME	Iran	Native Qazvin
KR059533-35	ME	Iran	Kuhaki
KR059536-37	ME	Iran	Morkhoj
KR059538	ME	Iran	Kuhaki
KR059539-41	ME	Iran	Morkhoj
KR059542-43	ME	Iran	Kuhaki
KR059544-45	ME	Iran	Morkhoj
KR059546	ME	Iran	Kuhaki
KR059547	ME	Iran	Morkhoj
KR059548	ME	Iran	Kuhaki
KR059550-52	ME	Iran	Morkhoj
KR059553	ME	Iran	Kuhaki
KR059556	ME	Iran	Kuhaki
KR059557	ME	Iran	Morkhoj
KR059558	ME	Iran	Kuhaki
KR059559	ME	Iran	Morkhoj

KR059603-05	ME	Jordan	Baladie
KR059616-23	ME	Saudi Arabia	Beshi
KR059624-27	ME	Saudi Arabia	Najrani
KR059660-64	ME	Turkey	Abaza
KR059665-68	ME	Turkey	Angora
KR059670-74	ME	Turkey	Angora
KR059675-81	ME	Turkey	Gurku
KR059682-89	ME	Turkey	Hair
KR059704-18	ME	Iran	Khalkhali
KR059733-38	ME	Iran	Morkhoj
KR059750	ME	Iran	Naini
EF617863-67	ME	Iran	ND
EF617868	ME	Iran	Morkhoj
EF617869	ME	Iran	ND
EF617870-71	ME	Iran	Morkhoj
EF617872	ME	Iran	ND
EF617873	ME	Iran	Morghoz
EF617874	ME	Iran	NS
EF617875	ME	Iran	Morghoz
EF617876-901	ME	Iran	ND
EF618191-204	ME	Jordan	Baladi
EF617902-74	ME	Iran	ND
EF617975-8001	ME	Iran	Kurdi
EF618003-07	ME	Iran	Kurdi
EF618008-16	ME	Iran	Indeterminate breed
EF618017-56	ME	Iran	Raini
EF618057-67	ME	Iran	Indeterminate breed
EF618068-69	ME	Iran	Morghoz
EF618309-14	ME	Saudi Arabia	Beeshi
EF618315-21	ME	Saudi Arabia	Najrani
EF618322-30	ME	Saudi Arabia	Beeshi
EF618331-40	ME	Saudi Arabia	Najrani
EF618492-96	ME	Turkey	Abaza
EF618497	ME	Turkey	Angora goat
EF618498-504	ME	Turkey	Gurcu
EF618505-06	ME	Turkey	Hair
EF618507-14	ME	Turkey	Abaza
EF618515-22	ME	Turkey	Angora goat
EF618523-26	ME	Turkey	Gurcu
EF618527-34	ME	Turkey	Hair
KR059751-64	ME	Iran	Naini
KR059765-70	ME	Iran	Najdi
KR059771-76	ME	Iran	Raini
KR059816-17	ME	Iran	Taleshi
KR059820-28	ME	Iran	Taleshi
KR059829-40	ME	Iran	Turki
KR059842-43	ME	Iran	Turki
AJ317777-79	NAF	Algeria	ND

AJ317780-83	NAF	Egypt	ND
AJ317795-801	NAF	Egypt	ND
AJ317784-88	NAF	Morocco	ND
AJ317810-11	NAF	Nigeria	ND
AJ317823-25	NAF	Nigeria	ND
AJ317789-94	NAF	Tunisia	ND
AJ317816-18	NAF	Senegal	ND
KR059155	NAF	Egypt	Zaraibi
KR059157	NAF	Spain	Zaraibi
KR059159	NAF	Egypt	Zaraibi
KR059188	NAF	Egypt	Zaraibi
KR059227-28	NAF	Egypt	Barki
KR059229	NAF	Egypt	Zaraibi
KR059230	NAF	Egypt	Barki
KR059231	NAF	Egypt	Zaraibi
KR059232	NAF	Egypt	Barki
KR059233	NAF	Egypt	Zaraibi
KR059234	NAF	Egypt	Barki
KR059235	NAF	Egypt	Zaraibi
KR059236	NAF	Egypt	Barki
KR059237	NAF	Egypt	Zaraibi
KR059238	NAF	Egypt	Barki
KR059239	NAF	Egypt	Zaraibi
KR059240	NAF	Egypt	Barki
KR059241	NAF	Egypt	Zaraibi
KR059242	NAF	Egypt	Zaraibi
KR059243	NAF	Egypt	Barki
KR059244-51	NAF	Egypt	Zaraibi
KR059252	NAF	Egypt	Barki
KR059271-304	NAF	Egypt	Barki
EF617711-26	NAF	Egypt	Baladi
EF618220	NAF	Libya	ND
EF618233	NAF	Morocco	Moroccocan Black
EF618246-52	NAF	Nigeria	West African Dwarf
KR059777	NAF	Egypt	Saidi
KR059779-813	NAF	Egypt	Saidi
KR059815	NAF	Egypt	Saidi
KR059844-51	NAF	Egypt	Zaraibi
KR059778	NAF	Egypt	Saidi
AJ317650	NE	Denmark	ND
AJ317592	NE	England	ND
AJ317841	NE	England	ND
AJ317573-74	NE	Switzerland	ND
AJ317596-99	NE	Switzerland	ND
AJ317605	NE	Switzerland	ND
AJ317619-24	NE	Switzerland	ND
AJ317760-61	NE	Switzerland	ND
AJ317600-04	NE	Ukraine	ND

AJ317655-58	NE	Wales	ND
AJ317575-83	NE	France	ND
AJ317713-19	NE	France	ND
AJ317723-25	NE	France	ND
AJ317629-30	NE	France	ND
AJ317586-649	NE	Germany	ND
AJ317587-91	NE	Iceland	ND
AJ317593-95	NE	Norway	ND
AJ317584-85	NE	Poland	ND
AJ317651-52	NE	Poland	ND
AJ317653-54	NE	Slovakia	ND
AJ317731	NE	Slovenia	ND
AJ317637	NE	Sweden	ND
AJ317626	NE	Switzerland	ND
AJ317631-35	NE	Switzerland	ND
AJ317638-48	NE	Switzerland	ND
KR059147	NE	Switzerland	Valais Black Neck
KR059150	NE	Switzerland	Grisons Striped
KR059172	NE	Switzerland	Peacock goat
KR059181	NE	Switzerland	Swiss Alpine
KR059185	NE	France	Alpine
KR059194	NE	Germany	Thuringian Forest Goat
KR059342-54	NE	Austria	Pinzgau
KR059355-64	NE	Austria	Tauern Pied
KR059365-71	NE	Switzerland	Swiss Alpine
KR059372-76	NE	Switzerland	Grisons Striped
KR059377-83	NE	Switzerland	Peacock goat
KR059384-92	NE	Switzerland	St Gallen Booted goat
KR059393-97	NE	Switzerland	Valais Black Neck
KR059398-407	NE	Germany	German Alpine goat
KR059408-23	NE	Germany	Thuringian Forest Goat
KR059424	NE	France	Alpine
KR059426-29	NE	France	Alpine
KR059431-32	NE	France	Alpine
KR059433-43	NE	France	Corse
KR059444-57	NE	France	Pireneene
KR059458-72	NE	France	Rove
KR059606-10	NE	Poland	Brava
EF617710	NE	Denmark	Wallis
EF617729	NE	England	Kielder
EF618542	NE	Wales	Ramshill Isack
EF618543	NE	Wales	Wild goat
EF618544	NE	Wales	ND
EF618540	NE	Ukraine	Orienberg
EF617851	NE	Iceland	Kjarni
EF617852	NE	Iceland	Iceland Landrace
EF617853	NE	Iceland	Denso
EF617854	NE	Iceland	Stjaa

EF617855	NE	Iceland	Hafdis
EF618085-86	NE	Ireland	Irish Island
EF618346	NE	Slovenia	Bovska
EF618415-22	NE	Sweden	Suede Landrace
EF617601	NE	Austria	Pinzgau
EF617679-81	NE	Austria	Pinzgau
EF617682-84	NE	Austria	Tauern Pied
EF617685-91	NE	Austria	Pinzgau
EF617692-99	NE	Austria	Tauern Pied
EF617700	NE	Austria	Pinzgau
EF617730-31	NE	France	Rove
EF617732-37	NE	France	French Alpine
EF617738-42	NE	France	Corse
EF617743-45	NE	France	Pyrene
EF617746-51	NE	France	Rove
EF617752-54	NE	France	Poitou Poitevine
EF617755-61	NE	France	Corse
EF617762-69	NE	France	Pyrene
EF617770-77	NE	France	Rove
EF617778-85	NE	France	French Alpine
EF618423-24	NE	Switzerland	Grison Striped
EF618425-27	NE	Switzerland	Peacock Goat
EF618428	NE	Switzerland	Saanen
EF618429	NE	Switzerland	Valais blackneck
EF618430-34	NE	Switzerland	Toggenburg
EF618435-42	NE	Switzerland	Suiss Alpine
EF618443-48	NE	Switzerland	Grison Striped
EF618449-54	NE	Switzerland	Peacock Goat
EF618455-56	NE	Switzerland	St. Gallen booted
EF618457-61	NE	Switzerland	Suiss Alpine
EF618462-69	NE	Switzerland	Grison Striped
EF618470-77	NE	Switzerland	Peacock Goat
EF618478-83	NE	Switzerland	St. Gallen booted
EF618484-85	NE	Switzerland	Suiss Alpine
EF617788-94	NE	Germany	German Alpine
EF617795-807	NE	Germany	Thuringian Forest
EF617809-15	NE	Germany	German Alpine
EF618264-70	NE	Poland	Brava
EF618271-86	NE	Poland	Buk
MH489263-74	SA	India	Attapady
MH489322-23	SA	India	Attapady
MH489315-21	SA	India	Bidri
KP671449-56	SA	India	Kodi Adu
MH489305-13	SA	India	Berari
JX271775-78	SA	India	Zalawadi
KC818086-8105	SA	India	Zalawadi
MH489241-54	SA	India	Zalawadi
JX271772-74	SA	India	Surti

JX271768-69	SA	India	Surti
JX271763-66	SA	India	Surti
JX271750-62	SA	India	Sirohi
KC818063-85	SA	India	Sirohi
KX274337-46	SA	India	Sirohi
JX271739-49	SA	India	Sangamneri
KX274357-66	SA	India	Sangamneri
JX271729-38	SA	India	Osmanabadi
KC818028-38	SA	India	Osmanabadi
KX274347-56	SA	India	Osmanabadi
JX271727-28	SA	India	Mehsana
KC818011-27	SA	India	Mehsana
JX271717-26	SA	India	Marwari
KC817992-8010	SA	India	Marwari
JX271711-15	SA	India	Malabari
JX271707-09	SA	India	Malabari
KC817976-91	SA	India	Malabari
JX271701-06	SA	India	Kutchi
JX271697-99	SA	India	Kutchi
JX271700	SA	India	Kutchi
JX271685-96	SA	India	Kanniadu
JX271676-78	SA	India	Jamunapari
JX271666-73	SA	India	Jamunapari
KX274367-76	SA	India	Jamunapari
MH489430	SA	India	Jamunapari
MH489427-28	SA	India	Jamunapari
MH489420-25	SA	India	Jamunapari
KP671321-61	SA	India	Jamunapari
JX271674	SA	India	Jamunapari
MH489426	SA	India	Jamunapari
MH489410-19	SA	India	Jakhrana
JX271651-63	SA	India	Gohilwadi
KC817924-47	SA	India	Gohilwadi
MH489396-402	SA	India	Gohilwadi
MH489387-94	SA	India	Gohilwadi
JX271649-50	SA	India	Ganjam
JX271643-47	SA	India	Ganjam
KC817907-23	SA	India	Ganjam
JX271640-42	SA	India	Gaddi
KC817883-906	SA	India	Gaddi
JX271637-39	SA	India	Chegu
MH489355-66	SA	India	Chegu
JX271627-36	SA	India	Changthangi
KC817863-82	SA	India	Changthangi
JX271620-25	SA	India	Black Bengal
JX271617-18	SA	India	Black Bengal
JX271612-13	SA	India	Black Bengal
KC817843-62	SA	India	Black Bengal

DQ521881-86	SA	India	Black Bengal
DQ521879-80	SA	India	Black Bengal
DQ521871-78	SA	India	Black Bengal
JX271614	SA	India	Black Bengal
JX271616	SA	India	Black Bengal
JX271604-11	SA	India	Beetal
JX271601-02	SA	India	Beetal
KC817830-42	SA	India	Beetal
JX271599-600	SA	India	Barbari
JX271587-97	SA	India	Barbari
KC817816-23	SA	India	Barbari
KC817825-29	SA	India	Barbari
AB162213	SA	India	Barbari
AB110583-84	SA	India	Barbari
KX274327-34	SA	India	Barbari
EF617856-62	SA	India	Ragestan
AJ317856-57	SA	India	ND
AJ317540-44	SA	India	ND
AJ317560-62	SA	India	ND
AJ317571-72	SA	India	ND
AY155674-81	SA	India	ND
AY155683-707	SA	India	ND
AY155709-833	SA	India	ND
AY155835-79	SA	India	ND
AY155881-82	SA	India	ND
AY155884-951	SA	India	ND
AY155953-63	SA	India	ND
AY155966-93	SA	India	ND
AY155995-6008	SA	India	ND
AY156010-39	SA	India	ND
MG923212-26	SA	India	ND
MG923228-45	SA	India	ND
MG923247-48	SA	India	ND
MG923250-58	SA	India	ND
MG923260-66	SA	India	ND
MG923268-71	SA	India	ND
MG923273-95	SA	India	ND
MG923297-98	SA	India	ND
MG923300-15	SA	India	ND
AJ317533	SA	Pakistan	ND
AJ317539	SA	Pakistan	ND
AJ317554-55	SA	Pakistan	ND
AJ317557-59	SA	Pakistan	ND
AJ317563-65	SA	Pakistan	ND
AJ317845-50	SA	Pakistan	ND
AJ317861-63	SA	Pakistan	ND
AB110552-53	SA	Pakistan	Tapri
AB110557-58	SA	Pakistan	Patri

AB110560-63	SA	Pakistan	Pak Angora
AB110565-66	SA	Pakistan	Nachi
AB110567-69	SA	Pakistan	Lehri
AB110570	SA	Pakistan	Long Hairy
AB110572-74	SA	Pakistan	Kohistani
AB110575-78	SA	Pakistan	Khurawsani
AB110579	SA	Pakistan	Kamori
AB110580-82	SA	Pakistan	Dera Din Panah
AB110583-84	SA	Pakistan	Barbari
AB110585-88	SA	Pakistan	Beetal
AB110589	SA	Pakistan	Tapri
EF618253	SA	Pakistan	Beetal
EF618254	SA	Pakistan	Dera Dinpanah
AJ317851-55	SA	Bhutan	ND
AJ317804-09	SAF	Mozambique	ND
AJ317812-15	SAF	South Africa	ND
AJ317819-22	SAF	South Africa	ND
AJ317802-03	SAF	Zimbabwe	ND
EF618240-41	SAF	Mozambique	Pafuri
EF618242	SAF	Namibia	Kaoko
EF618243	SAF	Namibia	Owambo
EF618244	SAF	Namibia	Kaoko
EF618245	SAF	Namibia	Owambo
EF618351	SAF	South Africa	ND
EF618352	SAF	South Africa	Tsawana
EF618353-54	SAF	South Africa	Venda
EF618545-46	SAF	Zimbabwe	Matabelle
AY961629	SE	Portugal	Serrana
AY961630-33	SE	Portugal	Algarvia
AY961634-43	SE	Portugal	Serpentina
AY961645	SE	Portugal	Bravia
AY961646	SE	Portugal	Serrana
AY961647-48	SE	Portugal	Bravia
AY961649-52	SE	Portugal	Algarvia
AY961653	SE	Portugal	Bravia
AY961654-55	SE	Portugal	Serpentina
AY961656-57	SE	Portugal	Bravia
AY961658	SE	Portugal	Algarvia
AY961659	SE	Portugal	Serrana
AY961660	SE	Portugal	Serpentina
AY961661	SE	Portugal	Bravia
AY961662	SE	Portugal	Charnequeira
AY961663-64	SE	Portugal	Serpentina
AY961665-67	SE	Portugal	Bravia
AY961668	SE	Portugal	Serrana
AY961669-70	SE	Portugal	Serpentina
AY961671-73	SE	Portugal	Bravia
AY961674-75	SE	Portugal	Algarvia

AY961676-79	SE	Portugal	Charnequeira
AY961680	SE	Portugal	Serrana
AY961681	SE	Portugal	Bravia
AY961682	SE	Portugal	Algarvia
AY961683-86	SE	Portugal	Charnequeira
AY961687-93	SE	Portugal	Serrana
AY961694	SE	Portugal	Bravia
AY961695	SE	Portugal	Serpentina
AY961696-97	SE	Portugal	Bravia
AY961700	SE	Portugal	Charnequeira
AY961701-02	SE	Portugal	Serrana
AY961703	SE	Portugal	Charnequeira
AY961704-06	SE	Portugal	Serrana
AY961707	SE	Portugal	Bravia
AY961708-09	SE	Portugal	Serrana
AY961710-16	SE	Portugal	Bravia
AY961717	SE	Portugal	Algarvia
AY961718	SE	Portugal	Bravia
AY961719	SE	Portugal	Serpentina
AY961720	SE	Portugal	Bravia
AY961721-23	SE	Portugal	Algarvia
AY961724-25	SE	Portugal	Serpentina
AY961726	SE	Portugal	Algarvia
AY961727	SE	Portugal	Bravia
AY961728	SE	Portugal	Serrana
AY961729-30	SE	Portugal	Serpentina
AY961731-36	SE	Portugal	Bravia
AY961737-38	SE	Portugal	Serrana
AY961739	SE	Portugal	Algarvia
AY961740	SE	Portugal	Charnequeira
AY961741	SE	Portugal	Serrana
AY961742-44	SE	Portugal	Bravia
AY961745	SE	Portugal	Serrana
AY961746	SE	Portugal	Serpentina
AY961747	SE	Portugal	Charnequeira
AY961748-51	SE	Portugal	Serrana
AY961752-53	SE	Portugal	Serpentina
AY961754-57	SE	Portugal	Serrana
AY961758	SE	Portugal	Bravia
AY961759	SE	Portugal	Serpentina
AY961760	SE	Portugal	Serrana
AY961761	SE	Portugal	Serpentina
AY961762-63	SE	Portugal	Bravia
AY961764-66	SE	Portugal	Serrana
AY961767-68	SE	Portugal	Algarvia
AY961769-70	SE	Portugal	Serpentina
AY961771	SE	Portugal	Charnequeira
AY961772-73	SE	Portugal	Algarvia

AY961774	SE	Portugal	Serrana
AY961775-77	SE	Portugal	Bravia
AY961778-79	SE	Portugal	Serrana
AY961779	SE	Portugal	Serrana
AY961780-81	SE	Portugal	Algarvia
AY961782	SE	Portugal	Charnequeira
AY961783-84	SE	Portugal	Serrana
AY961785-86	SE	Portugal	Charnequeira
AY961787	SE	Portugal	Algarvia
AY961788-89	SE	Portugal	Serrana
AY961790	SE	Portugal	Bravia
AY961791-92	SE	Portugal	Serrana
AY961793	SE	Portugal	Bravia
AY961794	SE	Portugal	Serpentina
AY961795-99	SE	Portugal	Serrana
AY961800-801	SE	Portugal	Serpentina
AY961802	SE	Portugal	Algarvia
AY961803	SE	Portugal	Charnequeira
AY961804	SE	Portugal	Algarvia
AY961805	SE	Portugal	Charnequeira
AY961806-07	SE	Portugal	Serpentina
AY961808	SE	Portugal	Bravia
AY961809	SE	Portugal	Algarvia
AY961810-12	SE	Portugal	Bravia
AY961813-15	SE	Portugal	Serpentina
AY961816	SE	Portugal	Algarvia
AY961817	SE	Portugal	Serpentina
AY961818-19	SE	Portugal	Serrana
AY961820-21	SE	Portugal	Bravia
AY961822-23	SE	Portugal	Algarvia
AY961824-27	SE	Portugal	Serpentina
AY961828	SE	Portugal	Charnequeira
AY961829-30	SE	Portugal	Bravia
AY961831	SE	Portugal	Serpentina
AY961832	SE	Portugal	Serrana
AY961833	SE	Portugal	Bravia
AY961834-35	SE	Portugal	Charnequeira
AY961836	SE	Portugal	Serrana
AY961837-38	SE	Portugal	Bravia
AY961839	SE	Portugal	Charnequeira
AY961840	SE	Portugal	Serpentina
AY961841	SE	Portugal	Algarvia
AY961842	SE	Portugal	Serpentina
AY961843-47	SE	Portugal	Serrana
AY961848-49	SE	Portugal	Serpentina
AY961850-51	SE	Portugal	Serrana
AY961852-54	SE	Portugal	Serpentina
AY961855-58	SE	Portugal	Serrana

AY961859	SE	Portugal	Bravia
AY961860-61	SE	Portugal	Algarvia
AY961862-69	SE	Portugal	Serrana
AY961870-71	SE	Portugal	Algarvia
AY961872-74	SE	Portugal	Charnequeira
AY961875-77	SE	Portugal	Bravia
AY961878	SE	Portugal	Charnequeira
AY961879-81	SE	Portugal	Serrana
AY961882-83	SE	Portugal	Bravia
AY961884-87	SE	Portugal	Charnequeira
AY961888-89	SE	Portugal	Serrana
AY961890	SE	Portugal	Algarvia
AY961891	SE	Portugal	Charnequeira
AY961892-95	SE	Portugal	Algarvia
AY961896-97	SE	Portugal	Charnequeira
AY961898-99	SE	Portugal	Bravia
AY961900	SE	Portugal	Serrana
AY961901-02	SE	Portugal	Bravia
AY961903-06	SE	Portugal	Serrana
AY961907	SE	Portugal	Bravia
AY961908-09	SE	Portugal	Serrana
AY961910	SE	Portugal	Algarvia
AY961911	SE	Portugal	Serpentina
AY961912	SE	Portugal	Charnequeira
AY961913	SE	Portugal	Algarvia
AY961914	SE	Portugal	Serrana
AY961915-16	SE	Portugal	Serpentina
AJ317660-69	SE	Portugal	ND
AJ317698-701	SE	Portugal	ND
AJ317720-22	SE	Portugal	ND
AJ317726-30	SE	Portugal	ND
AJ317732-35	SE	Portugal	ND
AJ317606-18	SE	Romania	ND
DQ241305-48	SE	Sicily	ND
DQ241352-71	SE	Sicily	ND
AJ317774-76	SE	Cyprus	ND
AJ317686-97	SE	Greece	ND
AJ317674-78	SE	Italy	ND
AJ317680-85	SE	Italy	ND
AJ317659	SE	Malta	ND
AJ317702	SE	Malta	ND
AJ317708	SE	Malta	ND
AJ317625	SE	Spain	ND
AJ317670-73	SE	Spain	ND
AJ317679	SE	Spain	ND
AJ317703	SE	Spain	ND
AJ317704-07	SE	Spain	ND
AJ317709-12	SE	Spain	ND

KR059146	SE	Albania	Mati
KR059148	SE	Italy	Camosciata
KR059149	SE	Spain	Verata
KR059156	SE	Spain	Payoya
KR059162-64	SE	Cyprus	Cyprus
KR059165	SE	Italy	Girgentana
KR059166	SE	Albania	Muzhake
KR059168-69	SE	Spain	Verata
KR059170	SE	Albania	Muzhake
KR059173	SE	Spain	Verata
KR059174	SE	Romania	Carpathian
KR059176-77	SE	Italy	Girgentana
KR059182	SE	Spain	Verata
KR059187	SE	Romania	Carpathian
KR059190	SE	Spain	Payoya
KR059193	SE	Albania	Mati
KR059195	SE	Greece	Skopelos
KR059196	SE	Italy	Bionda
KR059197	SE	Italy	Orobica
KR059198	SE	Romania	Carpathian
KR059205	SE	Italy	ND
KR059209	SE	Italy	Grigia Molisana
KR059307-08	SE	Italy	Girgentana
KR059309-17	SE	Albania	Capore
KR059318-24	SE	Albania	Liquenasi
KR059325-34	SE	Albania	Mati
KR059335	SE	Albania	Muzhake
KR059336-41	SE	Albania	Muzhake
KR059473-81	SE	Greece	Skopelos
KR059482-501	SE	Italy	Capra di Montecristo
KR059561-71	SE	Italy	Argentata
KR059572-73	SE	Italy	Bionda
KR059574	SE	Italy	Camosciata
KR059575-85	SE	Italy	Girgentana
KR059586-98	SE	Italy	Grigia Molisana
KR059599	SE	Italy	Orobica
KR059600-01	SE	Italy	Sarda
KR059602	SE	Italy	Valdostana
KR059611-15	SE	Romania	Carpathian
KR059628	SE	Spain	Malaguena
KR059629-44	SE	Spain	Payoya
KR059645-59	SE	Spain	Verata
KR059697-702	SE	Italy	Garganica
KR059719-32	SE	Italy	Val di Livo
KR059739-49	SE	Italy	Nera di Verzasca
EF618296-300	SE	Romania	Carpathian
EF618301-08	SE	Romania	Austria Romania
EF618287	SE	Portugal	Ribatejana

EF618288-95	SE	Portugal	Transmontana
EF618232	SE	Malta	Alpine Malta
EF617707	SE	Cyprus	Machaeras
EF617816-19	SE	Greece	Indeterminate breed
EF617820-25	SE	Greece	Greek
EF617826-39	SE	Greece	Skopelos
EF617840-46	SE	Greece	Greek
EF617849	SE	Greece	Skopelos
EF618357	SE	Spain	Murcia Granada
EF618358-61	SE	Spain	Indeterminate breed
EF618362-65	SE	Spain	Florida
EF618366-71	SE	Spain	Guadarrama
EF618372-76	SE	Spain	Payoya
EF618377-80	SE	Spain	Verata
EF618381-88	SE	Spain	Florida
EF618389-96	SE	Spain	Guadarrama
EF618397-404	SE	Spain	Payoya
EF618405-12	SE	Spain	Verata
EF617601-06	SE	Albania	Capore
EF617607-12	SE	Albania	Dukati
EF617613-18	SE	Albania	Hasi
EF617619-20	SE	Albania	Liqenasi
EF617621-23	SE	Albania	Mati
EF617624-27	SE	Albania	Muzhake
EF617628-36	SE	Albania	Capore
EF617637-41	SE	Albania	Dukati
EF617642-49	SE	Albania	Hasi
EF617650-57	SE	Albania	Liqenasi
EF617658-64	SE	Albania	Mati
EF617665-72	SE	Albania	Muzhake
EF617673-74	SE	Albania	Dukati
EF617675	SE	Albania	Liqenasi
EF617676	SE	Albania	Mati
EF617677	SE	Albania	Liqenasi
EF618087-92	SE	Italy	Argentatadelletna
EF618093-99	SE	Italy	Biondadell Adamello
EF618100-02	SE	Italy	Camosciata Alpina
EF618103-08	SE	Italy	Girgentana
EF618109-12	SE	Italy	Grigia Molisana
EF618113-14	SE	Italy	Orobica
EF618115-20	SE	Italy	Sarda
EF618121-27	SE	Italy	Valdostana
EF618128-35	SE	Italy	Biondadell Adamello
EF618136-43	SE	Italy	Camosciata Alpina
EF618144-49	SE	Italy	Orobica
EF618150-57	SE	Italy	Valdostana
EF618158-65	SE	Italy	Grigia Molisana
EF618166-68	SE	Italy	Argentatadelletna

EF618169-76	SE	Italy	Girgentana
EF618177-81	SE	Italy	Argentatadelletna
EF618182-89	SE	Italy	Sarda
EF618190	SE	Italy	Argentatadelletna
AB044295-98	SEA	Laos	ND
AJ317553	SEA	Malaysia	ND
AJ317566-68	SEA	Vietnam	ND
MH760795	SEA	Indonesia	Boer
MH760792	SEA	Indonesia	Boer
AB440718-22	SEA	Myanamar	ND
AB440728	SEA	Myanamar	ND
AB750749-51	SEA	Philippines	ND
EF618221	SEA	Malaysia	Katjang
EF617702-05	WA	Azerbaijan	Azeri
EF617708-09	WA	Russia	Dagestani
EF618205-11	WA	Kazakhstan	Kazak
EF618212-16	WA	Kyrgyzstan	Kyrgyz
**Haplogroup B**			
MK139103, MK139108, MK139111-12, MK139117, MK139126-27, MT747080-100, MN865080, MN865082	ISEA	Andaman	Andaman local goat
AJ317833	EA	Mongolia	ND
AY860881	EA	China	ND
AY860888	EA	China	ND
AY860899	EA	China	ND
AY860901	EA	China	ND
AY860903	EA	China	ND
AY860918	EA	China	ND
AY860933	EA	China	ND
AY860924	EA	China	ND
AY860917	EA	China	ND
AY860914	EA	China	ND
AY860889	EA	China	ND
AY860922	EA	China	ND
AY860894	EA	China	ND
DQ188857	EA	China	ND
DQ188860	EA	China	ND
DQ188873	EA	China	ND
DQ188878	EA	China	ND
DQ188896	EA	China	ND
DQ121500	EA	China	ND
DQ121504	EA	China	ND
DQ121514	EA	China	ND

DQ121515-17	EA	China	ND
DQ121528-30	EA	China	ND
DQ121535	EA	China	ND
DQ121537-39	EA	China	ND
DQ121542	EA	China	ND
DQ121549	EA	China	ND
DQ121565-67	EA	China	ND
DQ121569-71	EA	China	ND
DQ121578-79	EA	China	ND
DQ121581	EA	China	ND
DQ121585	EA	China	ND
DQ121591	EA	China	ND
DQ121597-98	EA	China	ND
DQ121603	EA	China	ND
DQ121610-12	EA	China	ND
DQ121616	EA	China	ND
AY853287	EA	China	ND
AY853291-92	EA	China	ND
AY853294-95	EA	China	ND
AY853300-01	EA	China	ND
DQ089112	EA	China	ND
DQ089115	EA	China	ND
DQ089118	EA	China	ND
DQ089124	EA	China	ND
DQ089129	EA	China	ND
DQ089134	EA	China	ND
DQ089138	EA	China	ND
DQ089149-50	EA	China	ND
DQ089154	EA	China	ND
DQ089158-60	EA	China	ND
DQ089162-63	EA	China	ND
DQ089176	EA	China	ND
DQ089179	EA	China	ND
DQ089181	EA	China	ND
DQ089183-85	EA	China	ND
DQ089193	EA	China	ND
DQ089195-97	EA	China	ND
DQ089199	EA	China	ND
DQ089201	EA	China	ND
DQ089205	EA	China	ND
DQ089207	EA	China	ND
DQ089209	EA	China	ND
DQ089214-15	EA	China	ND
DQ089218	EA	China	ND
DQ089223	EA	China	ND
DQ089226-27	EA	China	ND
DQ089229	EA	China	ND
DQ089231	EA	China	ND

DQ089235	EA	China	ND
DQ089240-41	EA	China	ND
DQ089245	EA	China	ND
DQ089249	EA	China	ND
DQ089251	EA	China	ND
DQ089256-58	EA	China	ND
DQ089260-61	EA	China	ND
DQ089264	EA	China	ND
DQ089267-70	EA	China	ND
DQ089272-73	EA	China	ND
DQ089278-79	EA	China	ND
DQ089283	EA	China	ND
DQ089285	EA	China	ND
DQ089295-96	EA	China	ND
DQ089300-02	EA	China	ND
DQ089304-08	EA	China	ND
DQ089357-58	EA	China	ND
DQ089363-64	EA	China	ND
DQ089366	EA	China	ND
DQ089369	EA	China	ND
DQ089374	EA	China	ND
DQ089377-78	EA	China	ND
DQ089396	EA	China	ND
DQ089403-05	EA	China	ND
DQ089407-08	EA	China	ND
DQ089410-13	EA	China	ND
DQ089421	EA	China	ND
KR059219	ME	Iran	Bezoar
KR059305-06	ME	Iran	Bezoar
KR059703	ME	Iran	ND
KR059819	ME	Iran	Taleshi
EF618257	SA	Pakistan	Beetal
JX271770-71	SA	India	Surti
JX271767	SA	India	Surti
JX271747	SA	India	Sangamneri
JX271716	SA	India	Malabari
JX271710	SA	India	Malabari
JX271675	SA	India	Jamunapari
MH489429	SA	India	Jamunapari
JX271648	SA	India	Ganjam
JX271619	SA	India	Black Bengal
JX271603	SA	India	Beetal
KC817824	SA	India	Barbari
KX274335-36	SA	India	Barbari
AJ317827	SA	India	ND
AJ317830	SA	India	ND
AY155682	SA	India	ND
AY155834	SA	India	ND

MG923227	SA	India	ND
MG923272	SA	India	ND
MG923246	SA	India	ND
MG923249	SA	India	ND
MG923267	SA	India	ND
MG923296	SA	India	ND
MG923299	SA	India	ND
AY155880	SA	India	ND
AY155883	SA	India	ND
AY155994	SA	India	ND
AJ317826	SA	Pakistan	ND
AB110554	SA	Pakistan	Teddy
AB110556	SA	Pakistan	Teddy
AB110564	SA	Pakistan	Teddy
AB110571	SA	Pakistan	Long Hairy
EF618257	SA	Pakistan	Beetal
EF618258	SA	Pakistan	Nachi
EF61825	SA	Pakistan	Nachi
EF618260-63	SA	Pakistan	Teddy
AJ317844	SAF	South Africa	ND
EF618355-56	SAF	South Africa	Ciskei
EF617850	SE	Greece	Greek
EF618222	SEA	Malaysia	Katjang
AB044299	SEA	Laos	ND
AB044300-04	SEA	Laos	ND
AJ317828-29	SEA	Malaysia	ND
AJ317831-32	SEA	Malaysia	ND
MF048904	SEA	Indonesia	Samosir
MH760783-90	SEA	Indonesia	ND
MH760791	SEA	Indonesia	Boer
MH760793-94	SEA	Indonesia	Boer
AB440723-27	SEA	Myanamar	ND
AB440717	SEA	Myanamar	ND
AB750747-48	SEA	Philippines	ND
KR059220	SEA	Malaysia	Malaysia
EF618222-30	SEA	Malaysia	Katjang
EF617706	WA	Azerbaijan	Ajeri
**Haplogroup D**			
MK139131, MK139133-35, MK139138-39, MT747127-46	ISEA	Andaman	Teressa goat
KR059211-12	CA	Kyrgyzstan	Kyrgyzstan
DQ188888	EA	China	ND
DQ188893	EA	China	ND
DQ089350	EA	China	ND
KR059210	ME	Iran	Bezoar
EF617701	NE	Austria	Pinzgau

AY155964-65	SA	India	ND
AY155952	SA	India	ND
MH489395	SA	India	Gohilwadi
EF618217-19	WA	Kyrgyzstan	Kyrgyz

ND indicates no information on breed.

Table S3. AMOVA analysis of haplogroup A goats of Andaman and other different regions

Source of variation	d. f.	Sum of squares	Variance Components	Percentage of variation	Fixation Index FST	P-value
Among populations	9	12231.217	3.80198 Va	16.05	0.16052	0.00000±0.00000
Within populations	3914	77826.588	19.88416 Vb	83.95		
Total	3929	90057.805				

Table S4. AMOVA analysis of haplogroup B goats of Andaman and other different regions

Source of variation	d.f.	Sum of squares	Variance Components	Percentage of variation	Fixation Index FST	P-value
Among populations	7	110.980	0.56302 Va	25.01	0.25015	0.00000±0.00000
Within populations	266	448.939	1.68774 Vb	74.99		
Total	273	559.920	2.25077			

Table S5. AMOVA analysis of haplogroup D goats of Andaman and other different regions

Source of variation	d.f	Sum of squares	Varianace components	Percentage of variation	FST	P value
Among populations	6	9.413	0.38298 Va	84.35	0.84346	0.00000±0.00000
Within populations	34	2.417	0.07108 Vb	15.65		
Total	40	11.829	0.45406			

Table S6. Haplotype diversity of goat breeds

S. No.	Breed	Region	GenBank Accession nos.	No. of Haplotypes (h)	Haplotype Diversity (Hd ± SD)	Nucleotide Diversity (Pi ± SD)	Haplogroups
South Asia (SA)							
1	Andaman Local Goat	Andaman and Nicobar Islands	MK139101 - MK139130, MN865073 - MN865082, MT747030 - MT747126 (n=137)	35	0.897±0.011	0.01865±0.00093	A = 112<br>B = 25
2	Terresa Goat	Andaman and Nicobar Islands	MK139131 - MK139140, MT747127 - MT747164 (n=48)	4	0.621±0.052	0.01882±0.00104	A = 22<br>D = 26
3.	Barbari	UP and Rajasthan	JX271587-JX271597, JX271599-JX271600, KC817816 - KC817821, KC817823, KC817825-KC817829, KX274327-KX274334, KX274335-KX274336, AB110583 - AB110584, AB162213 (n = 41)	26	0.929±0.032	0.03695±0.00226	A = 39<br>B = 3
4	Black	West	JX271612-	37	0.979±0.011	0.027±0.0032	A =48

	Bengal	Bengal and Bangladesh	JX271614, JX271616- JX271619- JX271625, KC817843- KC817862, DQ521886 - DQ521871 (n=49)				B = 1
5	Changthang i	Jammu and Kashmir	JX271626- JX271636, KC817863- KC817882 (n=31)	28	0.991±0.012	0.02002±0.00375	A = 30 C = 1
6	Chegu	Himachal Pradesh	JX271637- JX271639, MH489355 - MH489366 (n=15)	14	0.990±0.028	0.01954±0.00250	A= 15
7	Gaddi	Himachal Pradesh	JX271640- JX271642, KC817883- KC817906 (n=27)	23	0.990±0.019	0.01554±.00226	A = 27
8	Ganjam	Orrisa	JX271643- JX271650, KC817907- KC817923 (n=25)	24	0.997±0.012	0.02284±0.00387	A = 25 B = 1
9	Gohilwadi	Gujrat	JX271651- JX271663, KC817924- KC817947, MH489387 - MH489402 (n=53)	48	0.996±0.004	0.02145±0.00166	A = 52 D = 1
10	Jakhrana	Rajasthan	MH489410 - MH489419 (n=10)	10	0.982±0.046	0.03069±0.004	A= 10
11	Jamunapari	Uttar Pradesh	JX271666- JX271678, KX274367- KX274376, MH489420	34	0.947±0.013	0.02456±0.00223	A = 73 B = 2

			- MH489430 , KP671321- KP671345, KP671347- KP671360 (n=73)				
12	Kanniadu	Tamil Nadu	JX271685- JX271696 (n=12)	5	0.667±0.141	0.00470±0.00182	A = 12
13	Kutchi	Gujarat	JX271697- JX271706 (n=10)	10	1.00±0.045	0.02356±0.00653	A = 10
14	Malabari	Kerala	JX271710- JX271716, KC817976- KC817990 (n=26)	17	0.960±0.022	0.02318±0.00431	A = 24, B = 2
15	Marwari	Rajasthan	JX271717- JX271726, KC817992- KC818010 (n=29)	22	0.975±0.017	0.01674±0.00124	A = 29
16	Mehsana	Gujarat	JX271728- JX271727, KC818011- KC818027 (n=19)	16	0.977±0.027	0.01865±0.00168	A = 19
17	Osmanabad i	Maharashtr a	JX271729- JX271738, KC818028- KC818038, KX274347- KX274356 (n=31)	17	0.880±0.051	0.0175±0.00143	A = 31
18	Sangamneri	Maharashtr a	JX271749, JX271745- JX271747, JX271739- JX271743, KX274357 KX274366 (n=19)	11	0.738±0.106	0.01683±0.00562	A = 18, B = 1
19	Sirohi	Rajasthan, Gujarat	JX271750- JX271762, KC818663- KC818685,	30	0.931±0.028	0.02291±0.00124	A = 46

			KX274337-KX274346 (n=46)				
20	Surti	Gujarat	JX271763-JX271774 (n=12)	8	0.924±0.057	0.3334±0.00564	A = 9, B = 3
21	Attapady	Kerala	MH489322-MH489323, MH489263-MH489274 (n=14)	14	1.00±0.027	0.02321±0.00538	A = 14
22	Beetal	Punjab	JX271601-JX271611, KC817830-KC817842, AB110585-AB110588, EF618253, EF618257 (n=30)	26	0.989±0.013	0.02866±0.00432	A = 28, B = 2
23	Berari	Maharashtra	MH489305-MH489314 (n=10)	10	0.982±0.046	0.04296±0.01356	A = 10, C = 1
24	Zalawadi	Gujarat	JX271775-JX271778, KC818086-KC818105, MH489241-MH489254 (n=38)	34	0.994±0.007	0.02111±0.00150	A = 38
25	Bidri	Karnataka	MH489315-MH489321 (n=7)	7	1.00±0.076	0.02605±0.01009	A = 7
26	Kodi Adu	Tamil Nadu	KP671449-KP671451, KP671453-KP671456 (n=7)	8	1.00 ± 0.063	0.01010±0.00136	A = 8
27	Ragestan	Rajasthan	EF617856-EF617862 (n=7)	7	1.000 ±0.076	0.34085±0.05290	A = 7

28	Dera Din Panah	Pakistan	EF618254-EF618255, AB110580-AB110582 (n=5)	4	0.900±0.161	0.01497±0.00327	A = 5
29	Khurawsani	Pakistan	AB110575-AB110578 (n=4)	4	1.00±0.177	0.00948±0.00191	A = 4
30	Kohistani	Pakistan	AB110572-AB110574 (n=3)	3	1.00±0.272	0.00621±0.00215	A = 3
31	Lehri	Pakistan	AB110568-AB110570 (n=3)	3	1.00 ±0.272	0.00869±0.00278	A = 3
32	Long Hairy	Pakistan	AB110570 AB110571, (n=2)	2	1.00±0.50	0.03818±0.01909	A = 1, B = 1
33	Nachi	Pakistan	AB110564-AB110566, EF618255, EF618258-EF618259 (n=6)	5	0.933±0.122	0.04047±0.00762	A = 3, B = 3
34	Pak Angaora	Pakistan	AB110560 -AB110563 (n=4)	4	1.00 ±0.177	0.01377±0.00292	A = 4
35	Patri	Pakistan	AB110557-AB110559 (n=3)	3	1.00 ±0.272	0.04035 ±0.01496	A = 2, C = 1
36	Tapri	Pakistan	AB110552 -AB110553 (n=2)	2	1.00±0.50	0.00495±0.00248	A = 2
37	Teddy	Pakistan	EF618260 -EF618263, AB110554-AB110556 (n=7)	4	0.714 ±0.181	0.02515±0.01542	B =5, C = 1
Middle East (ME)							
38	Bezoar	Iran	KR059210, KR059219, KR059221 - KR059222, KR059226 (n=5)	6	0.952±0.096	0.07523±0.01300	B = 1, C = 2 D = 1, F = 1
39	Kermansha h	Iran	KR059207 -	3	1.00±0.272	0.02090±0.00693	A= 3, G = 1

			KR059208, KR059189, KR059213 (n=4)				
40	khalkhali	Iran	KR059218, KR059704 - KR059718, KR059512 (n=17)	16	0.993±0.023	0.01366±0.00311	A= 16, G = 1
41	Kuhaki	Iran	KR059505, KR059524, KR059529, KR059533-KR059535, KR059538, KR059517, KR059542-KR059543, KR059546, KR059548, KR059553, KR059556, KR059558 (n=15)	13	0.981±0.031	0.01514±0.00091	A=15
42	Kurdi	Iran	KR059191, EF617975 - EF618001 - EF618003 - EF618007 (n=33)	26	0.985±0.011	0.02069±0.00138	A = 33
43	Marghoz	Iran	EF617865, EF617868, EF617870-EF617871, EF617873, EF617875, EF618068-EF618069 (n=27)	24	0.991±0.013	0.02133±0.00151	A = 27
44	Naini	Iran	KR059192, KR059750-KR059764, KR059525 (n=17)	17	1.00±0.020	0.01274±0.0078	A = 17
45	Najdi	Iran	KR059765-KR059770 (n=6)	6	1.00±0.096	0.01531±0.00182	A = 6

46	Native Abadeh	Iran	KR059253-KR059266 (n=14)	13	0.989±0.031	0.01171±0.00089	A = 14
47	Native Qazvin	Iran	KR059507, KR059514, KR059519, KR059521, KR059532, KR059527, KR059503 (n=7)	6	0.952±0.096	0.01386±0.00213	A = 7
48	Raini	Iran	EF618017 - EF618056 (n = 40)	41	0.995±0.006	0.02237±0.00095	A = 40
49	Taleshi	Iran	KR059504, KR059506, KR059508-KR059511, KR059515-KR059516, KR059522-KR059523, KR059816-KR059819, KR059820-KR059828 (n=23)	19	0.984 ±0.017	0.01587±0.00293	A= 21, B = 1, G = 1
50	Turki	Iran	KR059502, KR059506, KR059526, KR059528-KR059543, KR059520, KR059530 - KR059531, KR059513, KR059518 (n=24)	23	0.996± 0.013	0.02358 ±0.00391	A= 22, D = 1, G = 1
51	Hair	Turkey	KR059204, EF618527-EF618534, EF618505-EF618506, KR059158, KR059215 (n=13)	17	0.981±0.020	0.02127±0.00347	A= 20, G = 1

52	Gurku	Turkey	EF618523-EF618526, EF618535-EF618537, EF618498-EF618504, KR059161, KR059203 (n=16)	15	0.964 ±0.020	0.02516±0.00351	A= 13, G = 3
53	Angora	Turkey	KR059214, KR059202-KR059200, EF618497, EF618515-EF618522 (n=17)	20	0.998±0.016	0.02975±0.00361	A= 16, G = 1
54	Abaza	Turkey	KR059179, KR059186, KR059199, EF618492-EF618496, EF618507 - EF618514 (n=16)	21	0.976±0.023	0.01878±0.00218	A = 16
55	Najrani	Saudi Arabia	EF618331, EF618344-EF618345, EF618315-EF618321, EF618332-EF618340, KR059154 (n=20)	21	0.989±0.015	0.02752±0.00384	A= 18, G = 2
56	Beeshi	Saudi Arabia	KR059184, EF618309 - EF618314, EF618341-EF618343, EF618322 - EF618330 (n=19)	23	0.984±0.014	0.02367±0.00325	A= 16, G = 3
57	Baladi	Jordan	EF617727 - EF617728, EF618191-EF618204, KR059153, KR059160, KR059183	16	0.974±0.019	0.02576±0.00421	A= 17, G = 2

			(n=19)				
South East Asia (SEA)							
58	Boar	Indonesia	MH760791 - MH760795 (n=5)	5	1.000± 0.126	0.02265±0.00605	A = 2, B = 3
59	Katjang	Malaysia	EF618221-EF618230 (n=10)	5	0.818±0.083	0.01187±0.00683	A = 1, B = 9
Western Asia (WA)							
60	Azeri	Azerbaijan	EF617702 - EF617706 (n=5)	5	1.000±0.126	0.43448±0.14531	A = 4, B = 1
61	Dagestani	Russia-Dagestan	EF617708-EF617709 (n=2)	2	1.000±0.500	0.01663±0.00832	A = 2
62	Kazak	Kazakhstan	EF618205 - EF618211 (n=7)	6	0.952±0.096	0.01841±0.00326	A = 5
63	Kyrgyzstan	Kyrgyzstan	EF618212 - KR058216, KR059211-KR059212 (n=7)	7	1.000±0.076	0.40952±0.05979	A = 5, D = 2
